# Circular RNA circ_0000515 adsorbs miR-542-3p to accelerate bladder cancer progression via up-regulating ILK expression

**DOI:** 10.18632/aging.203818

**Published:** 2022-01-14

**Authors:** Guohui Peng, Jing Guan, Pengfei Leng, Lijun Peng, Manchao Cao, Yuanfa Feng

**Affiliations:** 1 Department of Urology, BenQ Medical Center, The Affiliated BenQ Hospital of Nanjing Medical University, Nanjing 210019, China; 2 Department of Geriatric and Cardiology, Qingdao Fuwai Hospital, Qingdao 266034, China; 3 Department of Urology, Qingdao Fuwai Hospital, Qingdao 266034, China; 4 Department of Ultrasound, Qingdao Fuwai Hospital, Qingdao 266034, China

**Keywords:** bladder cancer, circ_0000515, miR-542-3p, ILK

## Abstract

Background: Bladder cancer (BC) is a common cause of cancer-relevant deaths globally. This study is designed to delve into expressions, biological functions and molecular mechanisms of circ_0000515 in BC.

Methods: Quantitative real-time polymerase chain reaction was accomplished to examine circ_0000515, miR-542-3p and integrin-linked kinase (ILK) mRNA expressions in BC tissues and cell lines. In RT-4 and RT-112 cells with circ_0000515 depletion and UMUC3 and BIU-87 cells with this circ RNA overexpression, a cell counting kit-8 assay was adopted to monitor the viability. Besides, transwell assay was conducted to detect cell migration and aggressiveness, and luciferase reporter gene assay was applied to probe the interplay among circ_0000515, miR-542-3p and ILK mRNA. Additionally, Besides, the regulatory function of circ_0000515 on miR-542-3p expression was under the assay of quantitative real-time polymerase chain reaction, and western blot was fulfilled to determine the regulative function of circ_0000515/miR-542-3p axis on ILK protein expressions. A xenograft animal was modeled to examine lung metastasis *in vivo*.

Results: Circ_0000515 and ILK expressions were significantly elevated in BC tissues and cell lines, while that of miR-542-3p was dramatically suppressed. Knocking down circ_0000515 could significantly repress the growth, migration and aggressiveness of BC cells while overexpression of circ_0000515 showed opposite effects. Moreover, circ_0000515 knockdown inhibited pulmonary metastasis *in vivo*. Circ_0000515 was validated to adsorb miR-542-3p, and ILK was testified as the downriver target of miR-542-3p. Circ_0000515 could ascend ILK expression through repressing that of miR-542-3p.

Conclusions: Circ_0000515, as a tumor promoter, strengthens the viability, migration and aggressiveness of BC cells via modulating miR-542-3p/ILK axis.

## INTRODUCTION

Bladder cancer (BC) is a prevalent driver of cancer-pertinent deaths globally. BC incidence rises with age, reaching a peak at 50-70 years old [1]. Despite the great progress in strategies for treatment, the prognostic index of BC patients is still pessimistic, and specifically, the relapse rate after surgery is as much as 70% within 5 years [2]. Therefore, the next focus on the progression course of BC molecular mechanism may ensure valuable insights into the treatment of this disease.

Circular RNAs (circRNAs) are recognized as non-coding RNAs with closed-loop structures and are pertinent to the progression of multiple diseases, including bladder cancer. For example, circ_0071196 facilitates BC cell proliferative and migrative abilities via tuning miR-19b-3p/CIT axis [3]; circRNA FOXO3 inhibits BC progression via regulating miR-9-5p/TGFBR2 axis [4]; circ_0061140 boosts BC metastasis via targeting miR-1236 [5]. Nonetheless, how circ_0000515 functions in BC is inconclusive.

MicroRNAs (miRNAs) are known as non-coding RNA with 18-24 nt in length. Reportedly, certain miRNAs are anomalously expressed in BC tissues/cells and are closely pertinent to the tumorigenesis and advancement of BC, such as miR-542-3p [6, 7]. Integrin-linked kinase (ILK) is conceptually a serine-threonine protein kinase and can control many biological behaviors of cells, including growth, survival, differentiation and migration [8]. Many studies show that ILK overexpression is associated with the carcinogenesis and metastasis of diverse cancers, such as hepatocellular carcinoma, colorectal cancer and BC [9–11].

CircRNAs can participate in tumor progression through competitive endogenous RNA (ceRNA) mechanism, that is, circRNAs adsorb miRNAs and regulate downstream target genes’ expressions [12]. For example, CircRNA FAM114A2 suppresses the malignant phenotypes of BC cells via modulating ∆NP63 and adsorbing miR-762 [13]. However, the ceRNA networks in BC has not been totally recognized.

Here we substantiated that circ_0000515 expressions were exceptionally elevated in BC tissues and cell lines, and was interrelated with the tumor size and clinical stage of the sufferers, and circ_0000515 could promote the malign and biologic behaviors of BC cells via regulating miR-542-3p/ILK axis. Our findings about this ceRNA network provided clues for BC diagnosis and treatment.

## RESULTS

### Circ_0000515 expressions were greatly elevated in BC tissues and cell lines

**Figure 1:**
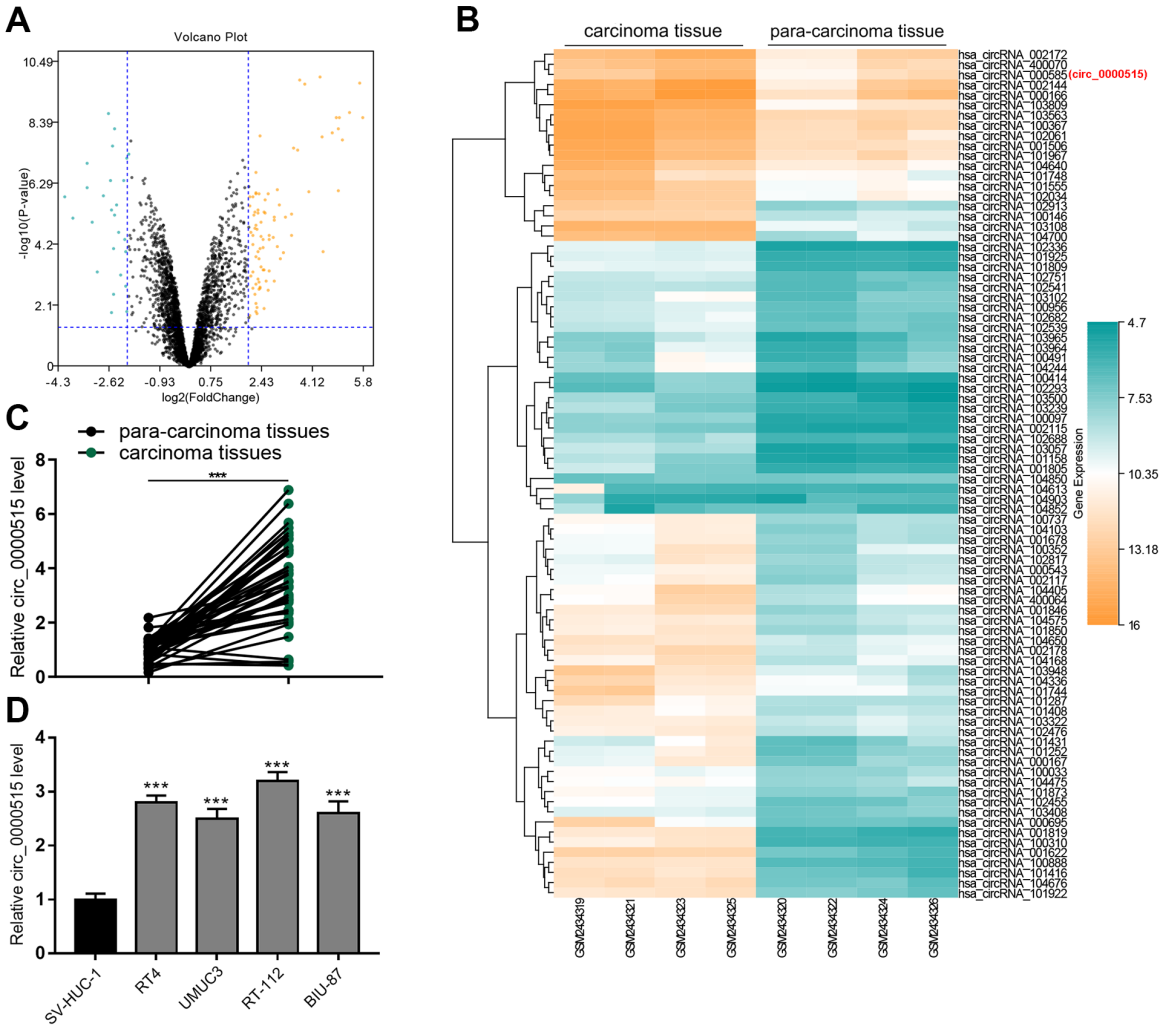
**Circ_0000515 expression was greatly raised in BC tissues and cell lines.** (**A**) Volcanic plot showed the differentially expressed circRNAs in BC tumor tissues and adjacent tissues. (**B**) Heat map showed some representative ascended circRNAs in BC tumor tissues. (**C**, **D**) qRT-PCR ensured the analysis of circ_0000515 expressions in BC tissues and cell lines.***P<0.001.

By analyzing the circRNA microarray data (GSE92675), the expression patterns of circRNAs in four pairs of BC/normal bladder tissues were investigated. (Figure 1A, 1B). As shown, there were multiple circRNAs with exceptionally ascended expression in BC tissues, and circ_0000515 (also known as circ_000585) was among them (Figure 1B). qRT-PCR uncovered that circ_0000515 expression in BC tissues was greatly higher than that in adjacent tissues, being consistently with bioinformatics analysis (Figure 1C). We also observed that circ_0000515 expression in BC cell lines was demonstrably higher than that in normal bladder epithelial cell lines, especially in RT-4 cells and RT-112 cells (Figure 1D). Interestingly, as shown in Table 1, the high expression of circ_0000515 was pertinent to larger tumor diameter and higher clinical stage of BC sufferers. Therefore, we hypothesized that circ_0000515 could probably be implicated in the BC progression as a tumor-promoter.

**Table 1:** Correlation between circ_0000515 levels and clinicopathological parameters.

**Parameter**	**N**	**circ_0000515**		***P***
		**High (n=20)**	**Low (n=20)**	
Age (years)				
≥ 60	23	11	12	0.749
< 60	17	9	8	
Gender				
Male	25	10	15	0.102
Female	15	10	5	
Tumor size (cm)				
≥ 3	15	11	4	0.022
<3	25	9	16	
Clinical stage				
III-IV	20	15	5	0.001
I-II	20	5	15	

### Circ_0000515 had a closed-loop structure, and circ_0000515 depletion could curb the malignant biologic processes of BC cells

**Figure 2:**
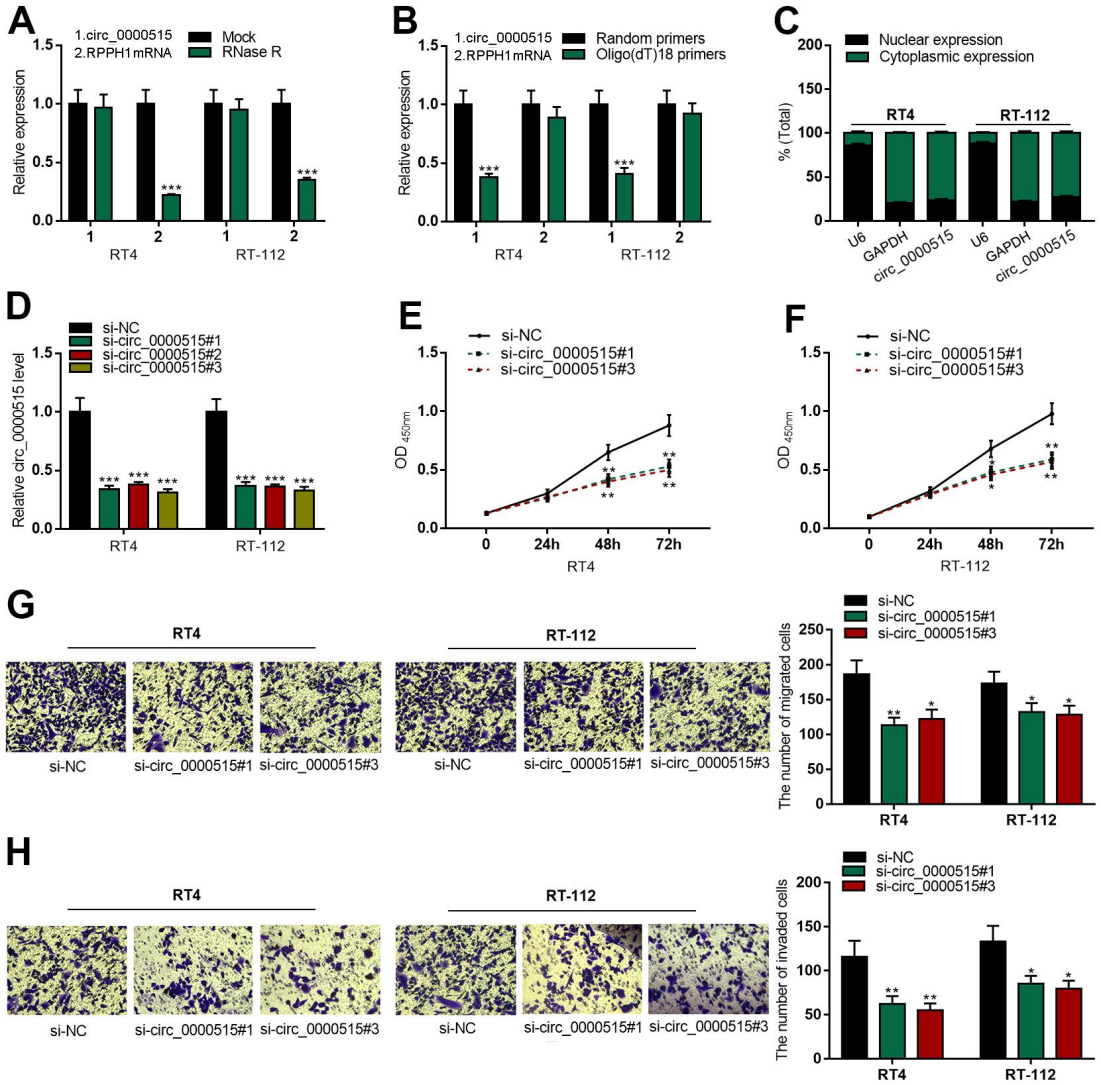
**Knocking down circ_0000515 restrained BC cell multiplication, migration and aggressiveness.** After the transfection of siRNA into BC cells: (**A**) qRT-PCR ensured the enrichment of circ_0000515 and RPPH1 mRNA in total RNA treated with RNase R. (**B**) qRT-PCR probed expressions of circ_0000515 and RPPH1 with Oligo(dT)18 primers. (**C**) Circ_0000515 expression in the cytoplasm and nucleus of BC cells was detected by qRT-PCR. (**D**) qRT-PCR was wielded to probe circ_0000515 expression. (**E**, **F**) CCK-8 assay was adopted to monitor the proliferation of BC cells. (**G**, **H**) Transwell assay displayed the migrative and invasive capabilities of BC cells. **P<0.01 and ***P<0.001.

To validate the circular structure of circ_0000515, we treated total RNA extracted from the cells with RNase R. As shown, linear RPPH1 mRNA (the precursor mRNA of circ_0000515) expression in RNase R group was significantly lower than that in Mock group, while no change of circ_0000515 expression was observed in both groups, and this suggested that circ_0000515 was resistant to RNase R (Figure 2A, 2B). Circ_0000515 expression in Oligo (dT)18 primers group was exceptionally lower than that in random primers group, while linear RPPH1 mRNA had no change between the two sets, displaying that circ_0000515 had no poly(A) tail. To illuminate the subcellular localization of circ_0000515, we probed circ_0000515 expressions in the cytoplasm and nucleus of BC cells, respectively, and observed that circ_0000515 was mainly located in cytoplasm (Figure 2C), implying that circ_0000515 could assumably work as a ceRNA in BC.

Next, we transfected three kinds of circ_0000515 siRNA into RT4 and RT-112 cells and found that circ_0000515 expressions in BC cells were decreased significantly after the transfection, among which si-circ_0000515#1 and si-circ_0000515#3 with the highest transfection efficiency were selected for the follow-up assays (Figure 2D). Compared with si-NC group, CCK-8 assay revealed that the cell multiplication in si-circ_0000515#1 and si-circ_0000515#3 groups was demonstrably inhibited (Figure 2E, 2F). Transwell assay indicated that the ability of cell migration and invasion in si-circ_0000515#1 and si-circ_0000515#3 groups were exceptionally lower than that in si-NC group (Figure 2G, 2H).

### Overexpression of circ_0000515 could promote the biological behaviors of BC cells

**Figure 3:**
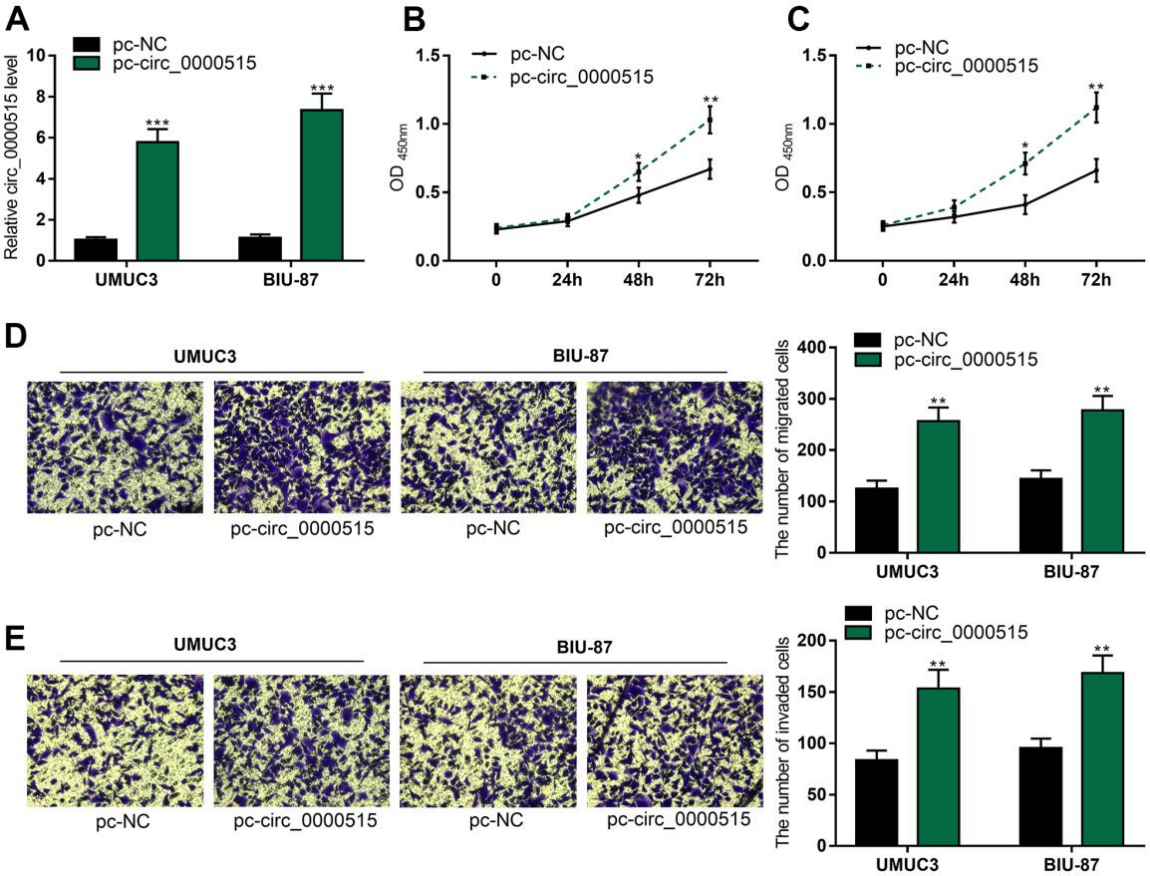
**Overexpression of circ_0000515 expedited BC cell proliferation, migration and aggressiveness.** After the transfection of overexpressing plasmid into BC cells: (**A**) qRT-PCR assay exposed circ_0000515 expressions. (**B**, **C**) The multiplication of BC cells was under the determination of CCK-8 assay. (**D**, **E**) Transwell assay ensured the analysis on migration and invasion of BC cells. **P<0.01 and ***P<0.001.

Then, we overexpressed circ_0000515 in UMUC3 and BIU-87 cells by the transfection with circ_0000515 overexpression plasmid (Figure 3A). As CCK-8 assay showed, circ_0000515 overexpression greatly enhanced UMUC3 and BIU-87 cell viability (Figure 3B, 3C). In addition, Transwell assay revealed that the migration and aggressiveness of UMUC3 and BIU-87 cells were markedly increased after circ_0000515 overexpression (Figure 3D, 3E). Collectively, circ_0000515 could promote the malignant biological processes of BC cells.

### Circ_0000515 was a molecular sponge of miR-542-3p

**Figure 4:**
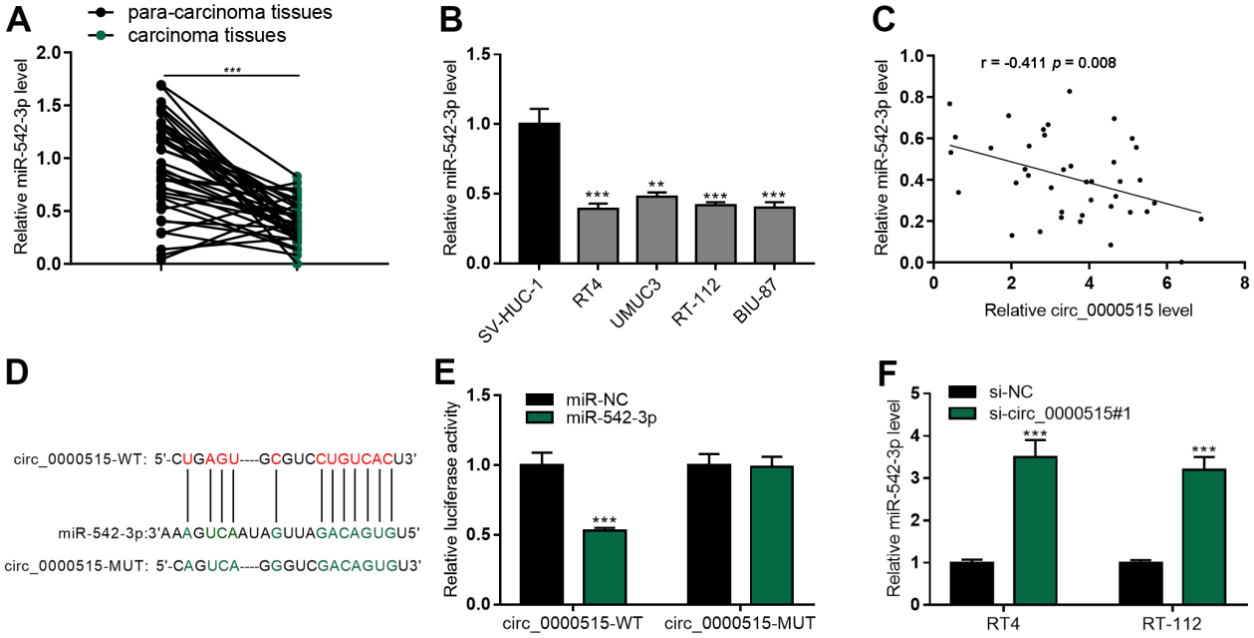
**MiR-542-3p was the molecular sponge of circ_0000515.** (**A**, **B**) qRT-PCR assay exposed miR-542-3p expression in BC tissues and cell lines. (**C**) The correlation analysis of miR-542-3p and circ_0000515 expression in BC tissue. (**D**) StarBase database predicted the complementary binding site between circ_0000515 sequence and miR-542-3p. (**E**) Luciferase reporter gene assay confirmed that miR-542-3p and circ_0000515 could interact with each other. (**F**) miR-542-3p expressions in BC cell lines transfected with circ_0000515 siRNA were under the detection of qRT-PCR. *P<0.05, **P<0.01 and ***P<0.001.

qRT-PCR uncovered that miR-542-3p expressions in BC tumor tissues and cell lines was exceptionally lower, as against that in normal tissues / cell line (Figure 4A, 4B), and miR-542-3p expressions were in negative correlation with circ_0000515 expressions (Figure 4C). StarBase database highlighted that circ_0000515 sequence harboured a binding site complementary to miR-542-3p (Figure 4D). Luciferase reporter gene assay confirmed that miR-542-3p mimics could significantly reduce the activity of circ_0000515-WT reporter, but that of circ_0000515-MUT reporter was not greatly impacted (Figure 4E). Moreover, miR-542-3p expression in BC cell line with circ_0000515 depletion was increased significantly (Figure 4F). These findings indicated that miR-542-3p was the downriver target of circ_0000515.

### Circ_0000515 modulated ILK expressions through miR-542-3p

**Figure 5:**
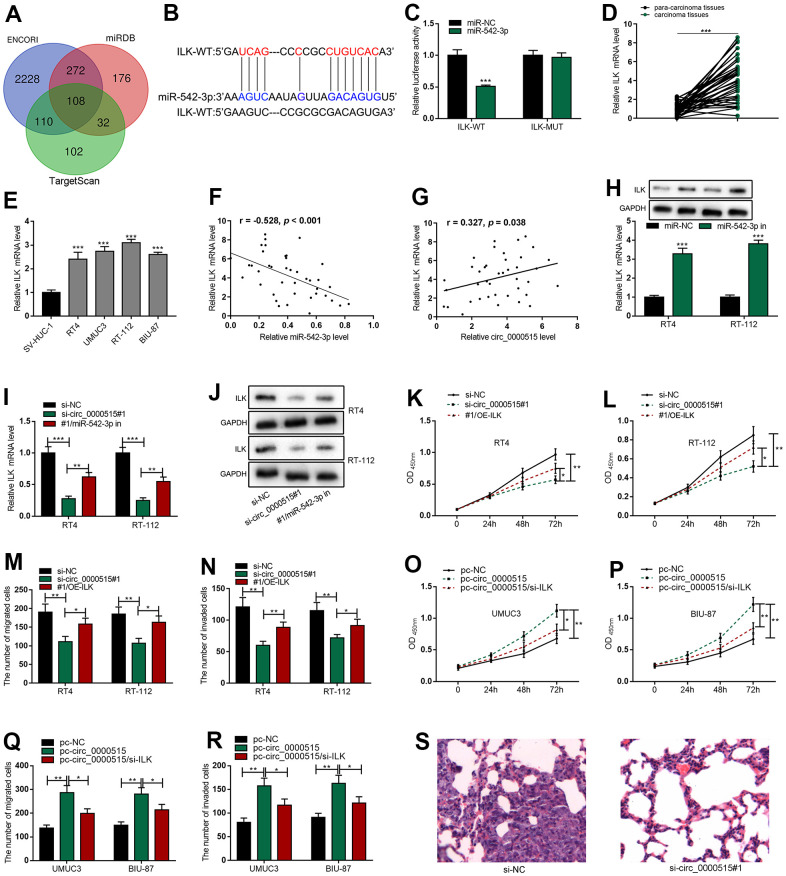
**Circ_0000515 regulated ILK expressions through miR-542-3p.** (**A**) Venn diagram screened the candidate mRNAs containing complementary binding sites with miR-542-3p. (**B**) StarBase database predicted that ILK 3’UTR harbored the binding site complementary to miR-542-3p. (**C**) Dual-luciferase reporter gene assay confirmed that miR-542-3p could directly bind to the 3’UTR of ILK. (**D**, **E**) ILK mRNA expressions in BC tissues and cell lines were detected by qRT-PCR. (**F**, **G**) The correlation analysis of miR-542-3p and ILK mRNA, and ILK mRNA and circ_0000515 in BC tissue. (**H**) qRT-PCR (upper) and western blot (below) assays exposed ILK expressions in BC cells transfected with miR-542-3p inhibitor. (**I**, **J**) qRT-PCR and western blot were used to probe ILK expressions in BC cells co-transfected with circ_0000515 siRNA and miR-542-3p inhibitor. (**K**, **L**) CCK-8 assay was applied to detect the multiplication of BC cells co-transfection with circ_0000515 siRNA and ILK overexpressing plasmid. (**M**, **N**) Transwell assay was exerted to probe the migration and aggressiveness of BC cells co-transfected with circ_0000515 siRNA and ILK overexpressing plasmid. (**O**, **P**) CCK-8 assay exposed the viability of BC cells co-transfected with circ_0000515 overexpressing plasmid and ILK siRNA. (**Q**, **R**) Transwell assay was wielded to detect the migration and invasion of BC cells co-transfected with circ_0000515 overexpressing plasmid and ILK siRNA. (**S**) The H&E staining was used to probe the lung metastasis of the mice, which were injected with RT4 cells transfected with si-circ_0000515#1 or si-NC, and the representative images were shown. *P<0.05, **P<0.01 and ***P<0.001.

To elaborate on the downstream target of miR-542-3p in bladder cancer, we searched StarBase database, TargetScan database and miRDB database, and 108 potential genes were predicted to have the complementary binding sites with miR-542-3p, and ILK was among the candidate targets (Figure 5A, 5B). Luciferase reporter gene assay showed that miR-542-3p mimics greatly reduced the activity of ILK-WT reporter but had no impact on that of ILK-MUT reporter (Figure 5C). We adopted qRT-PCR to detect ILK mRNA expressions in BC tissues and cell lines, and found that ILK mRNA expressions were significantly elevated in BC tissues and cell lines (Figure 5D, 5E). In addition, Table 2 detailed that the high expression of ILK was pertinent to the higher clinical stage of BC sufferers, suggesting it could probably be a cancer promoter in BC. Correlation analysis revealed that ILK mRNA expression was negatively correlated with miR-542-3p in BC tissues but positively with circ_0000515 (Figure 5F, 5G). Next, miR-542-3p inhibitor was transfected into BC cells, and qRT-PCR and western blot assays manifested that ILK mRNA and protein expression were ascended significantly; circ_0000515 knockdown repressed the expression level of ILK in both RT-4 and RT-112 cells while miR-542-3p inhibitors could partially reverse the inhibitory effects of knocking down circ_0000515 on ILK expressions (Figure 5H–5J and Supplementary Figure 1). These findings suggested that ILK was the downstream target of miR-542-3p in bladder cancer, and circ_0000515 regulated ILK expression through decoying miR-542-3p. We subsequently explored whether circ_0000515 regulated the malign behaviors of BC cells in an ILK-dependent manner. We co-transfected ILK overexpression plasmid and circ_0000515 siRNA into RT4 and RT-112 cells and found that ILK overexpression plasmid could significantly mitigate the inhibiting effects of circ_0000515 depletion on proliferative, migrative and invasive abilities of BC cells (Figure 5K–5N). Then, circ_0000515 overexpression plasmid and ILK siRNA were co-transfected into UMUC3 and BIU-87 cells, and the findings highlighted that ILK knockdown could rescue the promoting effects of circ_0000515 overexpression on the malignant processes of BC cells (Figure 5O–5R). Therefore, it was concluded that circ_0000515 regulated the malign behaviors of BC cells in an ILK-dependent manner.

**Table 2:** Correlation between ILK mRNA levels and clinicopathological parameters.

**Parameter**	**N**	**ILK**		***P***
		**High (n=20)**	**Low (n=20)**	
Age (years)				
≥60	23	13	10	0.337
< 60	17	7	10	
Gender				
Male	25	11	14	0.327
Female	15	9	6	
Tumor size (cm)				
≥ 3	15	10	5	0.102
< 3	25	10	15	
Clinical stage				
III-IV	20	14	6	0.011
I-II	20	6	14	

### Knockdown of circ_0000515 inhibited the lung metastasis of BC *in vivo*

To further validate the oncogenic function of circ_0000515 in BC, a nude mice model with lung metastasis was established. RT4 cells transfected with si-circ_0000515#1 or si-NC were injected into the tail vein of the nude mice, and 4 weeks later, H&E staining of lung tissue sections uncovered that, in the control, 9 of the 10 mice developed obvious lung metastasis, while in circ_0000515 knockdown group, only 2 of the 10 mice developed lung metastasis, suggesting that circ_0000515 promoted the lung metastasis of BC *in vivo* (Figure 5S).

## DISCUSSION

CircRNAs, as reported, are pivotal regulators in human diseases and are considered as new biomarkers of tumors. Circ_0000515 can promote the progression of various tumors [14–16]. For example, circ_0000515 can adsorb miR-326 and promote the advancement of cervical cancer via up-regulating ELK1 expressions [14]; in breast cancer, reportedly, circ_0000515 participates in disease advancement via modulating miR-296-5p/CXCL10 axis [15]; circ_0000515 promotes the malignancy of hepatocellular carcinoma cells via decreasing MAPK10 expression [16]. Nonetheless, how circ_0000515 functions in BC is unclear. Here we found that circ_0000515 expressions in BC tumor tissues were exceptionally higher than that in adjacent tissues, and it was associated with larger tumor diameter and higher clinical stage. In addition, circ_0000515 expression was also greatly ascended in BC cell lines as opposed to normal human bladder epithelial cell lines. Gain-of-function and loss-of-function assays confirmed that circ_0000515 could significantly promote the malign biologic behaviors of BC cells, highlighting that circ_0000515 could probably act as an oncogenic circRNA in the advancement of BC and be a hidden therapeutic target for BC.

Reportedly, there are ceRNA regulatory networks in tumors, and circ_0000515 can sponge miRNAs to promote the progression of tumors via regulating gene expressions post-transcriptionally [14, 15]. In this work, we confirmed that circ_0000515 had a circular structure and mainly distributed in the cytoplasm of BC cells. Bioinformatics analysis and luciferase reporter gene assay showed that miR-542-3p could directly bind to circ_0000515. As reported, miR-542-3p can inhibit the advancement of various tumors [17–20]. For example, miR-542-3p expressions are greatly down-regulated in esophageal cancer, and miR-542-3p can inhibit the migration and aggressiveness of esophageal cancer cells by inhibiting OTUB1 expression [17]; in osteosarcoma, miR-542-3p can curb cell proliferative, migrative and aggressive abilities via targeting ILK [18]; in ovarian cancer, miR-542-3p can target CDK14 to inhibit tumorigenesis [19]; in bladder cancer, importantly, miR-542-3p expression is significantly impaired, and this miRNA can inhibit the viability of BC cells [20]. Herein, we authenticated that miR-542-3p expression was demonstrably down-regulated in BC tissues and cell lines, and negatively pertinent to circ_0000515 expressions in BC tissues, and depleting circ_0000515 in BC cells significantly elevated miR-542-3p expressions, underlining that circ_0000515 might function via acting on miR-542-3p.

As is well known, miRNAs are implicated in tumorigenesis and development via targeting mRNAs. In the present study, luciferase reporter gene assay confirmed that miR-542-3p could bind to the 3'UTR of ILK mRNA, which was consistent with the bioinformatics analysis. ILK is conceptually a multifunctional intracellular effector of cell-matrix interaction, which can directly interact with β1 or β3 integrin subunit; ILK can coordinate various signal pathways, modulate cell proliferation by activating Akt signaling, and directly act on GSK-3β to activate some transcription factors [8]. In lung adenocarcinoma, as reported, ILK can regulate KRAS, IPP complex and Ras suppressor-1 (RSU1) to promote cancer cell multiplication, migration and epithelial-mesenchymal transition (EMT), and its high expression is pertinent to poor prognosis [21]. In glioma, ILK promotes cancer cell migration and aggressiveness via activating ROCK1 and fascin-1 [22]. Importantly, in bladder cancer, ILK expression is exceptionally up-regulated, which can expedite the malign biological behaviors of cancer cells [23]. Here we observed that ILK mRNA expression was dramatically elevated in BC tissues and cell lines, and was negatively pertinent to miR-542-3p expression and positively to circ_0000515 expression. Inhibiting miR-542-3p could significantly increase ILK expression, and miR-542-3p inhibitors could partially counteract the inhibiting effects of circ_0000515 knockdown on ILK expression. Besides, ILK overexpression could partially reverse the inhibiting effects of knocking down circ_0000515 on the malign biological behaviors of BC cells. Therefore, it was confirmed that circ_0000515 could adsorb miR-542-3p and ascend ILK expressions to boost BC advancement.

## CONCLUSIONS

Circ_0000515 promotes BC progression via targeting miR-542-3p to elevate ILK expression, highlighting that the ceRNA network composing of circ_0000515/miR-542-3p/ILK is a new mechanism of BC progression.

## MATERIALS AND METHODS

### Clinical sample collection

This work, with signed informed consents from the sufferers, was under the approval of the Ethics Committee of the Qingdao Fuwai Hospital. Tumor and paracancerous samples of 40 BC patients from the Qingdao Fuwai Hospital were gained during surgery and instantly preserved in liquid nitrogen at -196° C.

### Cell culture

Four BC cell lines (RT4, RT-112, UMUC3 and BIU-87) and human bladder epithelial cell line SV-huc-1 were available from the Chinese academy of sciences (Shanghai, China). The abovementioned cells were followingly cultured in Dulbecco's Modified Eagle Medium (Gibco, Carlsbad, CA, USA) with 10% fetal bovine serum (FBS; Gibco), penicillin (100 μg/ml, Gibco) and streptomycin (100 μg/ml, Gibco) at 37° C in 5% CO_2_.

### Cell transfection

Circ_0000515 overexpression plasmid, empty plasmid, circ_0000515 small interfering RNAs (siRNAs), a scrambled siRNA, miR-543-3p mimics, miR-542-3p inhibitors and their negative control miR-NC, ILK overexpressing plasmid and ILK siRNA, available from Genomeditech (Shanghai, China), were subsequently transfected into BC cell lines by Lipofectamine™ 2000 (Invitrogen, Carlsbad, CA, USA). After 72 h, the transfection efficiency was under the detection of quantitative real-time polymerase chain reaction (qRT-PCR) or western blots.

### Quantitative real-time polymerase chain reaction (qRT-PCR)

Total RNA was instantly extracted from BC tissues and cell lines by Trizol reagent (Life Technologies, Carlsbad, CA, USA), and 1 μg of RNA was synthesized into complementary DNA by Geneseed^®^ II First Strand cDNA Synthesis Kit. At last, SYBR Green Premix Ex Taq™ kit (Takara, Japan) was employed with glycerol dehyde-3-phosphate dehydrogenase (GAPDH) and U6 as internal references, and 2^-ΔΔCt^ was adopted to calculate RNA quantities. Primer sequences are listed in Table 3. To investigate the stability of circ_0000515, RNA samples were treated with 3 U/mg RNase R (Geneseed, Guangzhou, China) for 30 min, and circ_0000515 and RPPH1 mRNA expression levels were then accordingly quantified by qRT-PCR. To identify the subcellular localization of circ_0000515, a PARIS™ Kit (Invitrogen) was adopted for subcellular fractionation, and circ_0000515 expression in cytoplasm and nucleus of BC cells was respectively quantified by qRT-PCR, with U6 as the nuclear marker and GAPDH as the cytoplasmic marker.

**Table 3:** Primer sequences.

	**Forward primers**	**Reverse primers**
Circ_0000515	5’-GGTCAGACTGGGCAGGAGAT-3’	5’- GAGTGACAGGACGCACTCAG-3’
MiR-542-3p	5’-TCGGGGATCATCATGTCACG-3’	5’- GAGTGGCTCCCAGACCTTTC-3’
ILK	5’-GACGACATTTTCACTCAGTGCC-3’	5’- ACGGTTCATTACATTGATCCGTG-3’
RPPH1	5’ GTCACTCCACTCCCATGTCC-3’	5’-CAGCCATTGAACTCACTTCG-3’
U6	5’-GACTATCATATGCTTACCGT-3’	5’-GGGCAGGAAGAGGGCCTAT-3’
GAPDH	5’- TGACTTCAACAGCGACACCCA-3’	5’- CACCCTGTTGCTGTAGCCAAA-3’

### Cell proliferation assay

Cell counting kit-8 (CCK-8, Genomeditech, Shanghai, China) assay was employed to probe cell proliferation. Transfected BC cells were inoculated on a 96-well plate (5×10^3^ cells per well), and cultured for 0 h, 24 h, 48 h and 72 h. At each time point, 10 μL of CCK-8 reagent was dripped into each well to incubate the cells for 2 h. The value of OD_450nm_ was subsequently measured by a microplate reader (Thermo-Fisher Scientific, Waltham, MA, USA).

### Transwell assay

24-well Transwell chambers (8 μm pore size, Corning, Corning, NY, USA) were adopted to measure the migration and aggressiveness of BC cells. As to invasion assay, the filter was instantly covered with a layer of Matrigel, and Matrigel was not used in the migration assay. Bladder cells were inoculated into the upper chamber with 200 μL of serum-free medium; the lower was subsequently filled with RPMI-1640 medium with 10% FBS. After 24-h culture, the cells which passed through the filter were followingly stained with crystal violet solution, and then the number of cells was followingly counted by a microscope (Olympus, Tokyo, Japan).

### Dual-luciferase reporter assay

Circ_0000515 or ILK 3'UTR sequence harboring miR-542-3p binding site was subsequently amplified and then inserted into luciferase reporter plasmid (Promega, Madison, WI, USA) to obtain the wild type (WT) reporter, and the correspondent binding site of the above sequence was mutated and then the sequence was inserted into luciferase reporter plasmid to obtain the mutant type (MUT) reporter. Subsequently, the above luciferase reporters were instantly co-transfected into BC cells with miR-542-3p mimics or miR-NC by Lipofectamine™ 2000, and the activity was examined by the dual-luciferase reporter assay system (Promega) after 24 h.

### Western blot

The radio-immunoassay assay (RIPA) lysis buffer (Beyotime, Shanghai, China) was adopted to extract proteins from BC cells, which were quantified with a bicinchoninic acid (BCA) protein assay (Thermo-Fisher Scientific). Besides, protein samples were separated by sodium dodecyl sulfate-polyacrylamide gel electrophoresis and then accordingly transferred to polyvinylidene fluoride (PVDF) membranes (Millipore, Billerica, MA, USA), which were under the blockage of 5% skimmed milk and incubation with primary antibodies (anti-ILK: 1:1000, ab52480, Abcam Inc., Cambridge, UK; anti-GAPDH: 1:1000, ab8245, Abcam Inc.) overnight at 4° C and then with the horseradish peroxidase-conjugated secondary antibody (1: 2000, ab150077, Abcam Inc.) for 2 h, and an enhanced chemiluminescence kit (Biozym, Hessisch Oldendorf, Germany) ensured the development of protein bands, with GAPDH as the internal reference.

### Lung metastasis model *in vivo*

The Animal Care and Use Committee of the Qingdao Fuwai Hospital ensured the approval for all animal experiments. Notably, BALB/c nude mice (6 weeks old, male) was applied to model the lung metastasis. The mice were randomly grouped into two sets. In each, approximately 2×10^6^ RT4 cells transfected with si-circ_0000515#1 or si-NC were followingly injected into the tail vein of the nude mice (10 mice per group). 4 weeks later, all mice were instantly euthanized, and lung tissues were isolated. Hematoxylin and eosin (H&E) staining was used to detect lung metastasis.

### Statistical analysis

All assays were accomplished in triplicate, with findings expressed as “mean ± standard deviation”. The statistical analysis was tackled with SPSS (version 17.0) (SPSS Inc., Chicago, IL, USA). Student's *t*-test or one-way analysis of variance was wielded for the comparison of the data between/among groups, and Pearson’s correlation coefficient was applied for figuring out the correlation. Statistically, *P*<0.05 is meaningful.

## Supplementary Material

Supplementary Figure 1

## References

[1] Smith SG, Zaharoff DA. Future directions in bladder cancer immunotherapy: towards adaptive immunity. Immunotherapy. 2016; 8:351–65. 10.2217/imt.15.122 26860539 PMC5618954

[2] Lorenzatti Hiles G, Cates AL, El-Sawy L, Day KC, Broses LJ, Han AL, Briggs HL, Emamdjomeh A, Chou A, Abel EV, Liebert M, Palmbos PL, Udager AM, et al. A surgical orthotopic approach for studying the invasive progression of human bladder cancer. Nat Protoc. 2019; 14:738–55. 10.1038/s41596-018-0112-8 30683938 PMC6463286

[3] Liu Z, Yang Y, Yang Z, Xia S, Lin D, Xiao B, Xiu Y. Novel circRNA_0071196/miRNA-19b-3p/CIT axis is associated with proliferation and migration of bladder cancer. Int J Oncol. 2020; 57:767–79. 10.3892/ijo.2020.5093 32705161 PMC7384843

[4] Li Y, Qiao L, Zang Y, Ni W, Xu Z. Circular RNA FOXO3 Suppresses Bladder Cancer Progression and Metastasis by Regulating MiR-9-5p/TGFBR2. Cancer Manag Res. 2020; 12:5049–56. 10.2147/CMAR.S253412 32612392 PMC7323812

[5] Feng F, Chen AP, Wang XL, Wu GL. Circ_0061140 promotes metastasis of bladder cancer through adsorbing microRNA-1236. Eur Rev Med Pharmacol Sci. 2020; 24:5310–9. 10.26355/eurrev_202005_21313 32495864

[6] Catto JW, Alcaraz A, Bjartell AS, De Vere White R, Evans CP, Fussel S, Hamdy FC, Kallioniemi O, Mengual L, Schlomm T, Visakorpi T. MicroRNA in prostate, bladder, and kidney cancer: a systematic review. Eur Urol. 2011; 59:671–81. 10.1016/j.eururo.2011.01.044 21296484

[7] Wu HX, Wang GM, Lu X, Zhang L. miR-542-3p targets sphingosine-1-phosphate receptor 1 and regulates cell proliferation and invasion of breast cancer cells. Eur Rev Med Pharmacol Sci. 2017; 21:108–14. 28121348

[8] McDonald PC, Fielding AB, Dedhar S. Integrin-linked kinase--essential roles in physiology and cancer biology. J Cell Sci. 2008; 121:3121–32. 10.1242/jcs.017996 18799788

[9] Chan J, Ko FC, Yeung YS, Ng IO, Yam JW. Integrin-linked kinase overexpression and its oncogenic role in promoting tumorigenicity of hepatocellular carcinoma. PLoS One. 2011; 6:e16984. 10.1371/journal.pone.0016984 21347395 PMC3036736

[10] Almasabi S, Ahmed AU, Boyd R, Williams BR. A Potential Role for Integrin-Linked Kinase in Colorectal Cancer Growth and Progression via Regulating Senescence and Immunity. Front Genet. 2021; 12:638558. 10.3389/fgene.2021.638558 34163519 PMC8216764

[11] Zhuang X, Lv M, Zhong Z, Zhang L, Jiang R, Chen J. Interplay between intergrin-linked kinase and ribonuclease inhibitor affects growth and metastasis of bladder cancer through signaling ILK pathways. J Exp Clin Cancer Res. 2016; 35:130. 10.1186/s13046-016-0408-x 27576342 PMC5006283

[12] Chan JJ, Tay Y. Noncoding RNA:RNA Regulatory Networks in Cancer. Int J Mol Sci. 2018; 19:1310. 10.3390/ijms19051310 29702599 PMC5983611

[13] Liu T, Lu Q, Liu J, Xie S, Feng B, Zhu W, Liu M, Liu Y, Zhou X, Sun W, Zhang Y, Chen X, Fang F, et al. Circular RNA FAM114A2 suppresses progression of bladder cancer via regulating ∆NP63 by sponging miR-762. Cell Death Dis. 2020; 11:47. 10.1038/s41419-020-2226-5 31969560 PMC6976626

[14] Tang Q, Chen Z, Zhao L, Xu H. Circular RNA hsa_circ_0000515 acts as a miR-326 sponge to promote cervical cancer progression through up-regulation of ELK1. Aging (Albany NY). 2019; 11:9982–99. 10.18632/aging.102356 31772143 PMC6914414

[15] Cai F, Fu W, Tang L, Tang J, Sun J, Fu G, Ye G. Hsa_circ_0000515 is a novel circular RNA implicated in the development of breast cancer through its regulation of the microRNA-296-5p/CXCL10 axis. FEBS J. 2021; 288:861–83. 10.1111/febs.15373 32446265

[16] Li H, Li CM, Yuan R, Wang HB, Wei J. Circ_0000515 drives the progression of hepatocellular carcinoma by regulating MAPK10. Eur Rev Med Pharmacol Sci. 2020; 24:6014–22. 10.26355/eurrev_202006_21495 32572915

[17] Sun J, Deng Y, Shi J, Yang W. MicroRNA-542-3p represses OTUB1 expression to inhibit migration and invasion of esophageal cancer cells. Mol Med Rep. 2020; 21:35–42. 10.3892/mmr.2019.10836 31939620 PMC6896300

[18] Cai W, Xu Y, Zuo W, Su Z. MicroR-542-3p can mediate ILK and further inhibit cell proliferation, migration and invasion in osteosarcoma cells. Aging (Albany NY). 2019; 11:18–32. 10.18632/aging.101698 30636169 PMC6339804

[19] Li J, Shao W, Feng H. MiR-542-3p, a microRNA targeting CDK14, suppresses cell proliferation, invasiveness, and tumorigenesis of epithelial ovarian cancer. Biomed Pharmacother. 2019; 110:850–6. 10.1016/j.biopha.2018.11.104 30557834

[20] Zhang J, Wang S, Han F, Li J, Yu L, Zhou P, Chen Z, Xue S, Dai C, Li Q. MicroRNA-542-3p suppresses cellular proliferation of bladder cancer cells through post-transcriptionally regulating survivin. Gene. 2016; 579:146–52. 10.1016/j.gene.2015.12.048 26723509

[21] Nikou S, Arbi M, Dimitrakopoulos FD, Sirinian C, Chadla P, Pappa I, Ntaliarda G, Stathopoulos GT, Papadaki H, Zolota V, Lygerou Z, Kalofonos HP, Bravou V. Integrin-linked kinase (ILK) regulates KRAS, IPP complex and Ras suppressor-1 (RSU1) promoting lung adenocarcinoma progression and poor survival. J Mol Histol. 2020; 51:385–400. 10.1007/s10735-020-09888-3 32592097

[22] Louca M, Zaravinos A, Stylianopoulos T, Gkretsi V. ILK silencing inhibits migration and invasion of more invasive glioblastoma cells by downregulating ROCK1 and Fascin-1. Mol Cell Biochem. 2020; 471:143–53. 10.1007/s11010-020-03774-y 32506247

[23] Matsui Y, Assi K, Ogawa O, Raven PA, Dedhar S, Gleave ME, Salh B, So AI. The importance of integrin-linked kinase in the regulation of bladder cancer invasion. Int J Cancer. 2012; 130:521–31. 10.1002/ijc.26008 21351095

