# Supplementary material for: Circular RNA circ_0000515 adsorbs miR-542-3p to accelerate bladder cancer progression via up-regulating ILK expression
Source: Aging (Albany NY). 2022 Jan 14;14(1):430–42. doi: 10.18632/aging.203818 (PMC8791202; doi:10.18632/aging.203818)
Supplement: Supplementary Figure 1 [file aging-14-203818-s001.pdf]

SUPPLEMENTARY FIGURE

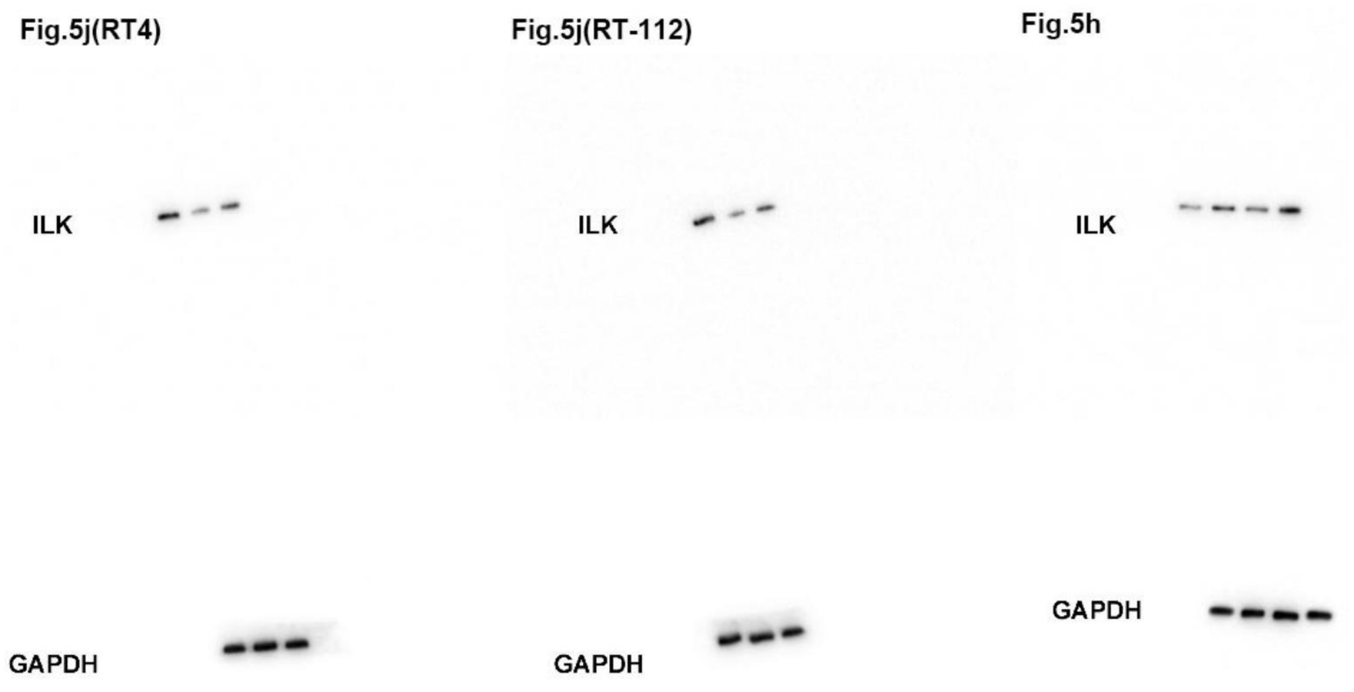

Supplementary Figure 1. The whole uncropped images of the original PVDF membranes in Figure 5.
